# Comparative quantification of focal and diffuse visual field loss by the SPARK Precision threshold algorithm and SITA

**DOI:** 10.1007/s00417-021-05430-7

**Published:** 2021-12-28

**Authors:** S. K. Foo, R. P. Cubbidge, R. Heitmar

**Affiliations:** 1 Faculty of Optometry and Vision Sciences, SEGi University, Petaling Jaya, Malaysia; 2 College of Health and Life Science, School of Optometry, Aston University, Birmingham, UK; 3 ABDO College, Godmersham Park, Canterbury, UK; 4 Department of Optometry and Vision Sciences, University of Huddersfield, Huddersfield, UK

**Keywords:** Perimetry, Visual field, SPARK Precision, SITA, Glaucoma, Cataract

## Abstract

**Purpose:**

The aims of this paper were to examine focal and diffuse visual field loss in terms of threshold agreement between the widely used SITA Standard Humphrey Field Analyser (HFA) threshold algorithm with the SPARK Precision algorithm (Oculus Twinfield 2).

**Methods:**

A total of 39 treated glaucoma patients (34 primary open angle and 5 primary angle closure glaucoma) and 31 cataract patients without glaucoma were tested in succession with the Oculus Twinfield 2 (Oculus Optikgeräte GmbH, Wetzlar, Germany) using the SPARK Precision algorithm and with the HFA 3 (Carl Zeiss Meditec, Dublin, CA) using the 30–2 SITA Standard algorithm.

**Results:**

SPARK Precision required around half the testing time of SITA Standard. There was a good correlation between the MS of the two threshold algorithms but MD and PSD were significantly less severe with SPARK Precision in both glaucoma (focal field loss) and cataract (diffuse field loss) groups (*p* < 0.001). There was poor agreement for all global indices (MS, MD and PSD) between the two algorithms and there was a significant proportional bias of MD in the glaucoma group and PSD in both glaucoma and cataract groups. The pointwise sensitivity analysis yielded higher threshold estimates in SPARK Precision than in SITA Standard in the nasal field. Classification of glaucoma severity using AGIS was significantly lower with SPARK Precision compared to SITA Standard (*p* < 0.001).

**Conclusion:**

SITA renders deeper defects than SPARK. Compared to the SITA Standard threshold algorithm, SPARK Precision cannot quantify early glaucomatous field loss. This may be due to the mathematical linear interpolation of threshold sensitivity or deeper scotomas due to the plateau effect caused by the reduced dynamic range of the Twinfield 2 perimeter. Although not of clinical significance in early glaucoma, the plateau effect may hinder the long-term follow-up of patients during disease progression.

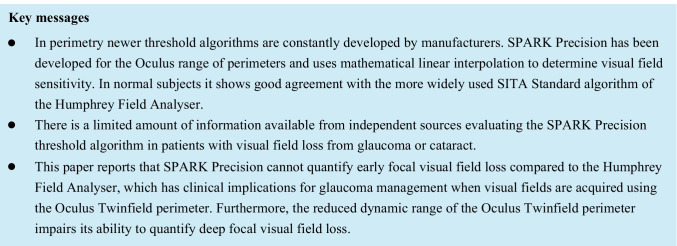



## Introduction

Visual field assessments are a core part of assessing patients with suspected or established visual field defects such as those suffering from glaucoma. In order for visual field tests to be used for clinical interpretation, they need to fulfil a number of criteria which are impacted by patient compliance, fixation, attention and fatigue [1–8]. In an attempt to eliminate and control such factors, new visual field algorithms have been developed some of which limit the number of test locations, time and others [9].

The threshold variability makes defining normality difficult when all tested locations are considered, meaning a patient can lose sensitivity for some time without exceeding the limits of what is considered statistically normal. In contrast, values outside normal limits do not guarantee a secure diagnosis of disease. Most clinical tests used for screening and diagnostic purposes have to fulfil specific criteria in respect to sensitivity and specificity, i.e. 95% specificity, which translates into obtaining false interpretations in 5% of the normal population. Simply introducing a higher cut-off criterion can reduce the number of false positives, but simultaneously reduces diagnostic capacity; hence, besides these criteria, there are often additional grading criteria to stratify patients into risk/disease groups [10, 11].

Diagnosis of glaucoma depends on a combination of factors including family history, intraocular pressure (IOP), optic nerve appearance and the visual field status [12].

New visual field algorithms, such as SPARK Precision (Oculus Optikgeräte GmbH, Wetzlar, Germany), have previously been shown to reduce the testing time [13] and provide comparable results in respect to mean sensitivities when compared to the widely used Humphrey Field Analyser (HFA) [13].

The aims of this paper were to examine focal visual field loss (commonly seen in glaucoma) and diffuse field loss (due to cataract) in terms of threshold agreement between the widely used SITA Standard (Humphrey Field Analyser, Carl Zeiss Meditec, Dublin, CA) threshold algorithm with the SPARK Precision algorithm (Oculus Twinfield 2, Oculus Optikgeräte GmbH, Wetzlar, Germany).

## Methods

A total of 39 treated glaucoma patients (34 primary open angle and 5 primary angle closure glaucoma) and 31 cataract patients without glaucoma were recruited from community eye clinics, hospitals and SEGi eye clinic (SEGi University, Malaysia). All patients had glaucoma/cataract diagnoses confirmed by an ophthalmologist. Each patient had established glaucoma which was previously diagnosed by an experienced ophthalmologist, based on the findings from contact tonometry, stereoscopic examination of the optic nerve head and visual field (VF) examination with 30–2 or 24–2 HFA SITA Standard VF testing. Cataract patients with variable levels of lenticular opacity were classified into type using the Lens Opacities Classification System (LOCS) III [14, 15] but with healthy fundi and best corrected visual acuity (BCVA) worse than 6/6.

Other inclusion criteria were absence of any ocular disease (other than glaucoma for the glaucoma group), intraocular pressure (IOP) ≤ 21 mmHg (for the cataract group), refractive errors below − 6.00DS/above + 6.00DS and less than 2.50DC astigmatism, no history of intraocular surgery or other ocular diseases that could affect the visual field. All participants were free from any systemic disease that could affect ocular health such as diabetes mellitus (DM) and hypertension (HT). Participants were asked to refrain from caffeine, alcohol and nicotine use for a minimum of 2 h prior to their examination. The study was approved by the Aston University Research Ethics Committee (ID 755) and adhered to the tenants of the Declaration of Helsinki.

### Data collection

Following written informed consent, all subjects underwent two visual field assessments on 2 separate days. At each visit, one randomly selected eye was tested in succession with the Oculus Twinfield 2 (Oculus Optikgeräte GmbH, Wetzlar, Germany) using the SPARK Precision algorithm and with the HFA (Carl Zeiss Meditec, Dublin, CA) using the 30–2 SITA Standard algorithm. The order of SITA and SPARK Precision testing was randomised among subjects to minimise order effects but remained constant between the two visits of a given individual.

Both instruments use a background luminance of 31.5 asb (10cdm^−2^) and have a maximum stimulus luminance of 10,000 asb. The 30–2 test pattern used in the HFA has a total of 76 test points covering the central 30° field with a square grid of 6° separation [16]. The SPARK Precision algorithm deploys a similar grid with a total of 66 test points (30° × 24°) with the uppermost and bottommost rows and two points located in the blind spots absent when compared to the SITA 30–2 test grid [9]. For the purpose of this study, all participants were examined with the four phases of the SPARK Precision algorithm. In brief, during the first phase, the 66 threshold values corresponding to the 66 test points are estimated by directly examining only six points, one in each functional region [17]. The six points are selected automatically using a stepwise multiple regression program, applied to a sample of 90,335 visual fields (derived using tendency oriented perimetry (TOP)), whereas two of the points are situated above and below the blind spot, another two are located in the superior and inferior nasal field and the remaining two points fall into the central region (below 10° eccentricity) and the temporal region. For example, the right eye coordinates for the six points could be (–15°, 15°), (15°, 15°), (–15°, –9°), (15°, 15°), (27°, –3°) and (3°, 3°). Both the two upper points and lower points are examined using an alternating bracketing strategy, utilising the response obtained from each to improve the estimated threshold of the other. The thresholds of the intermediate locations are then calculated by linear interpolation between those points corresponding to the same area. This results in all points being examined at least once. Based on these six locations, the sensitivities and deviations of the other locations are calculated using multiple regression analyses. The results of the first phase serve as a starting point for the successive estimates of threshold measures of the following three phases. During these three phases, threshold estimates are made of the 66 points by examining points situated in sectors corresponding to ganglion cell fibre bundles into which the glaucomatous visual field can be divided. These sectors largely coincide with those defined by functional analysis [18, 19]. Hence, in the three phases following phase one, these regions are examined using the threshold value estimated in the previous phase, and corrections are made with a magnitude equal to the associated previous standard error. In respect to number of points tested, 21 points (distributed regularly through all sectors) are examined in each of phase two to four, where the threshold of the intermediate points is subsequently calculated by linear interpolation between those points corresponding to the same area. When all four threshold estimates (one of each phase) have been obtained, their median value is calculated: which is equal to the average of the three closest threshold estimates, whereby the most extreme estimate has been disregarded; the latter aiming to reduce the influence of distraction or errors occurring in any of the four phases.

Participants were given a break of 10 min between the two tests. Previous research (our paper) showed good agreement between visits and between the two threshold methods; hence, for the purpose of this study, only results of the second visit were used to conduct between-strategy comparison because they are less influenced by the perimetric learning effect [17, 20]. All glaucoma patients included had undergone several perimetry tests previously, and hence were experienced, whereas only 18 of the cataract patients were experienced and the remainder was naïve.

### Visual field data analysis

Test results with poor reliability criteria, defined as false positives (FP) or negatives (FN) > 20% and fixation losses (FL) > 30% with SITA and FP > 20% and FL > 30% based on SPARK Precision, since it does not measure FN, were excluded from data analysis. A comparison of threshold variability within each of the SITA and SPARK strategies was made by carrying out a Bland–Altman analysis for the 66 stimulus locations in SPARK and the 66 stimulus locations in SITA which were coincident with the SPARK test pattern. To make a between-strategy comparison, the mean sensitivities of the 66 points test grid of SPARK and matching stimulus locations in the SITA test grid from the second visit were calculated for each patient and a pointwise analysis in between strategies was carried out to determine the threshold agreement between the two strategies. The results from the test points of the uppermost and bottommost rows of stimulus locations and the two points located at the blind spot for SITA were excluded as was the central foveal threshold in SPARK. For ease of analyses, the left eye results were transposed into right eye format.

The number of abnormal pattern deviation points (NAPDP) with at least *p* < 5% was also identified for each strategy, whereby the abnormal pattern deviation points for uppermost and bottommost test points in SITA Standard were not included.

### Further analyses of glaucoma patient data

The severity of VF defects among glaucoma patients from both strategies was determined and compared using the Advanced Glaucoma Intervention Study (AGIS) severity scale (The AGIS investigators, 1994). This scale is based on the number and depth of neighbouring depressed test locations on the total deviation plot of a single field analysis in the nasal area of the upper and lower hemifield. A test point is considered a depressed test location when a minimum deviation from normal is reached.

### Statistical analysis

Normality of the threshold data was determined by the Shapiro–Wilk test and the Wilcoxon signed ranks test which was used for between-strategy comparisons. Bland–Altman plots were used to determine the agreement of the mean sensitivities between both testing algorithms and regression testing was conducted to determine proportional bias. All correlations were determined using Spearman’s correlation coefficient. Statistical significance was set at a level of *p* < 0.05.

## Results

Out the 47 glaucoma patients recruited, we included for subsequent analysis a total of 39 glaucoma patients (six patients had been excluded as their refractive error fell outside the exclusion criteria and two were excluded as their reliability scores on both visits fell outside the exclusion criteria); 20 were female, and the distribution of field loss severity is detailed in Table 1. The same test order was used at both visits for each subject, where 21 patients were first tested with SITA Standard and the remainder with SPARK Precision.

**Table 1:** Severity of glaucoma according to AGIS score for glaucoma subjects

AGIS score	Category	No of patient	Percentage (based on SITA Standard)
0	None	16	41%
1–5	Mild	12	31%
6–11	Moderate	6	15%
12–17	Severe	5	13%
18–20	End-stage	0	0%

Among the 33 patients recruited, we excluded two (one due to missing the second appointment and one due to showing signs of diabetic retinopathy) cataract patients (18 females); 25 were classified with nuclear cataracts; three had both nuclear and cortical cataracts; and the remaining three had posterior subcapsular cataracts (LOCS III). Table 2 shows a summary of demographic data of both groups and Table 3 provides an overview of cataract patients’ best corrected visual acuities and cataract types.

**Table 2:** Age and spherical equivalent of glaucoma and cataract groups

	Glaucoma				Cataract			
	Mean	SD	Median		Mean	SD	Median	
Gender (M/F)	20/19							
Age (years)	54.7	12.6	55.0		60.8	9.6	63.0	
Spherical equivalent (D)	− 1.66	2.46	− 0.75		− 0.42	1.56	− 0.38

**Table 3:** Characteristics cataract patient

Best corrected visual acuity	*n*	Cataract type according to LOCS III
6/18	2	N
6/15	1	N
6/12	4	N
6/9	22	N, PSC, C
6/7.5	2	N

*N* nuclear; *PSC* posterior subcapsular; *C* cortical

The global indices and test duration in both groups were compared between the two thresholding methods (Table 4). There were statistically significant differences between the two threshold algorithms in respect to the mean deviation (MD), pattern standard deviation (PSD) and test time. The mean sensitivity (MS) of glaucoma patients appeared to yield larger numerical differences between the strategies when compared to the cataract group but the difference itself was not statistically significant. The MD obtained by SITA Standard showed more negative values than that of SPARK Precision in both the glaucoma and cataract groups. The PSD was distinctly higher with SITA Standard than with SPARK Precision in glaucoma patients but not in cataract patients. After taking into the consideration the difference in the number of test points (SPARK tests 10 locations less than SITA), the test time of SITA Standard was more than twice the time compared to SPARK Precision (Table 4; both patient groups).

**Table 4:** *MS* mean sensitivity; *MD* mean deviation; *PSD* pattern standard deviation; *NAPDP* number of abnormal pattern deviation points; *IQR* interquartile range. *Paired *t* test; showing mean and standard deviation rather than median and interquartile range

	Group	SITA Standard		SPARK Precision		*p* value
		Median	IQR	Median	IQR	*p*
MS (dB)	Glaucoma	27.36	11.2/31.0	28.42	11.6/32.3	0.451
	Cataract	27.18	13.5/30.3	27.97	17.3/31.0	0.003
MD (dB)	Glaucoma	− 2.31	− 16.7/0.8	0.32	− 11.1/ − 3.5	< 0.001
	Cataract	− 1.38	− 11.0/0.7	1.52	− 6.6/3.0	< 0.001
PSD (dB)	Glaucoma	4.85	1.5/14.8	1.76	0.9/8.1	< 0.001
	Cataract	2.07	1.1/8.2	1.51	0.8/3.4	< 0.001
Test time (min)	Glaucoma	7.27	5.5/12.2	3.55	3.3/3.9	< 0.001
	Cataract	7.64*	1.36*	3.67*	0.13*	< 0.001*
NAPDP	Glaucoma	14.0	1/47	8.0	0/35	0.035

## Agreement and correlation between strategies

### Mean sensitivity

The group MS from the matching 66 test points for both glaucoma and cataract groups were compared between threshold strategies. There were statistically significant correlations between the MS of SITA Standard and SPARK Precision in the glaucoma group (Spearman correlation coefficient: rho = 0.847, *p* < 0.001) and the cataract group (Spearman correlation coefficient: rho = 0.844, *p* < 0.001). Bland–Altman plots were used to determine the agreement of threshold estimates between the threshold strategies.

**Fig. 1:**
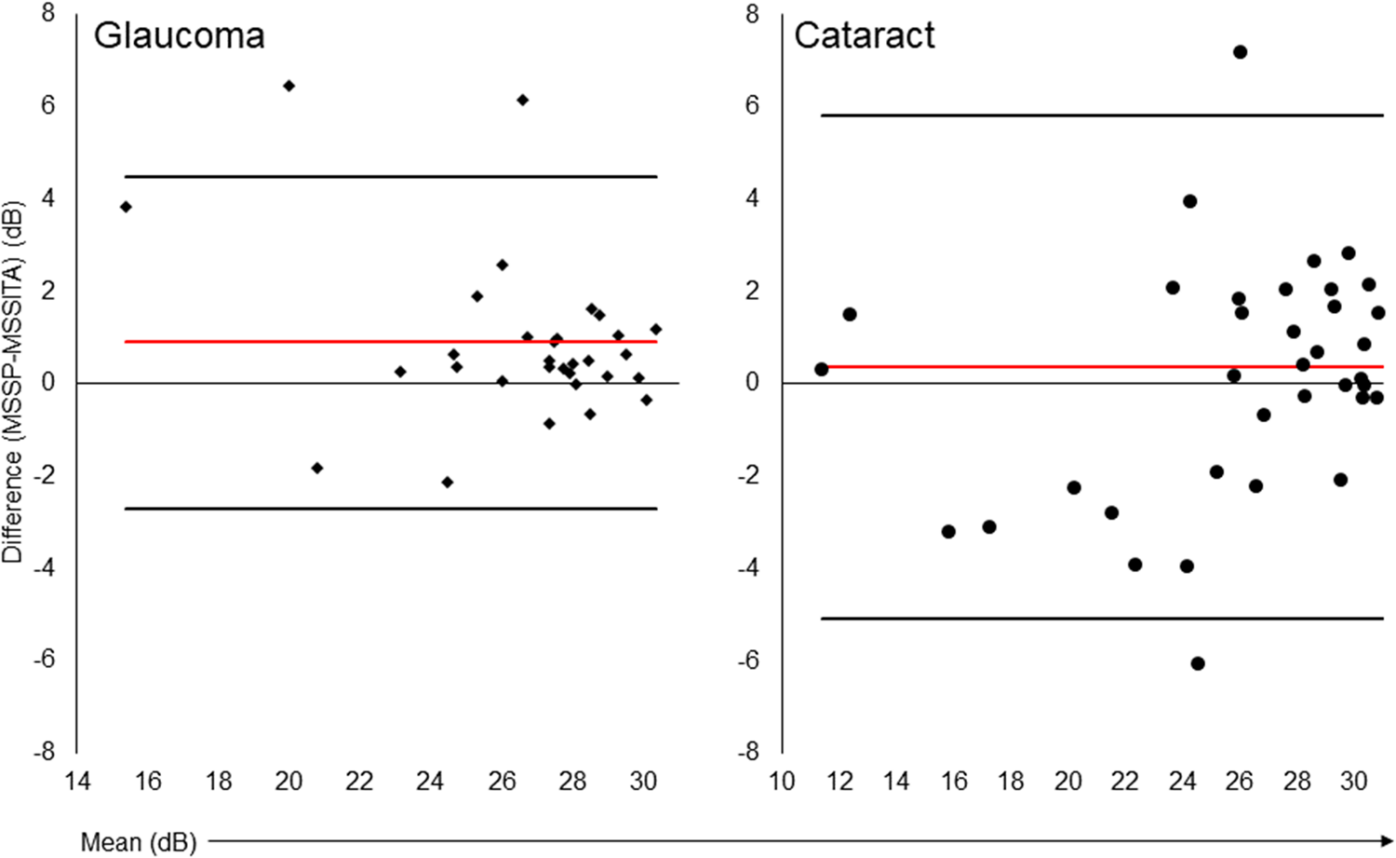
Bland–Altman plots of mean sensitivity between SS and SP in glaucoma and cataract patients. Red line denotes the bias, whereas the black lines denote the upper and lower LOA: limits of agreement

The Bland–Altman plot of MS of the glaucoma group is shown in Fig. 1. The bias/mean difference (95% LoA) between the threshold strategies was 0.37 dB (LoA − 5.08, 5.82 dB). There was no significant proportional bias for MS using regression (*t* = 0.888, *p* = 0.381).

In the cataract group, the bias was 0.90 dB (LoA − 2.69, 4.50 dB) with no proportional bias (*t* =  − 1.937, *p* = 0.062) (Fig. 1).

### Mean deviation

**Fig. 2:**
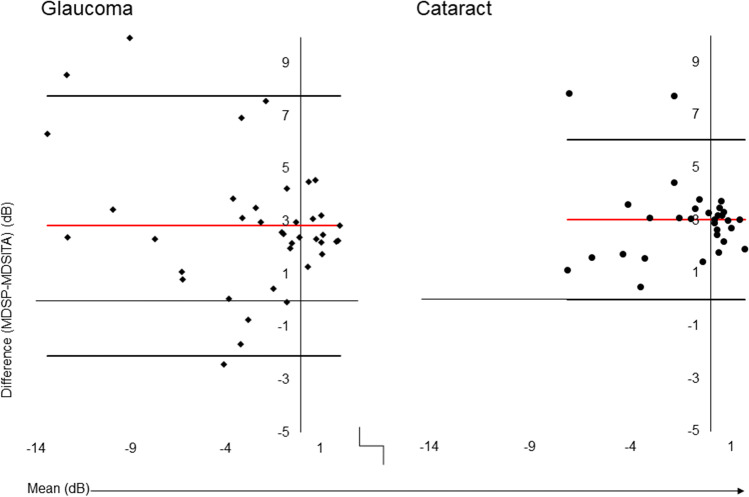
Bland–Altman plots of mean deviation between SS and SP in glaucoma and cataract patients. Red line denotes the bias, whereas the black lines denote the upper and lower LOA: limits of agreement

There was a lack of agreement of the MD between the threshold strategies in both groups as well as a significant proportional bias (*t* =  − 3.235, *p* = 0.02). The bias/mean difference (LoA) of MD between SITA Standard and SPARK Precision was 2.84 dB (LoA − 2.11 dB, 7.78 dB) in glaucoma patients and 3.03 dB (LoA − 0.01 dB, 6.06 dB) in cataract patients (see Fig. 2).

### Pattern standard deviation

**Fig. 3:**
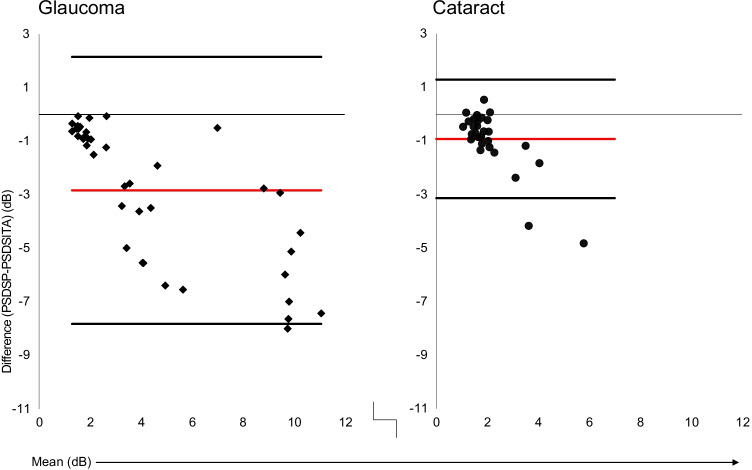
Bland–Altman plots of pattern standard deviation between SS and SP in glaucoma and cataract patients. Red line denotes the bias, whereas the black lines denote the upper and lower LOA: limits of agreement

Bland–Altman analyses for the PSD of SITA Standard and SPARK Precision of glaucoma patients yielded a mean bias of − 2.84 dB (LoA − 7.82, 2.15 dB). The PSD of SITA Standard yielded higher values compared to SPARK Precision (see Fig. 3). Regression testing showed a proportional bias (*t* =  − 7.302, *p* < 0.001) indicating larger differences between strategies as the severity of defect increases. In the cataract group, the mean bias (LoA) for PSD between threshold strategies was − 0.92 dB (LoA − 3.14 dB, 1.29 dB) and showed a proportional bias which was similar to that found in the glaucoma group (*t* =  − 8.101, *p* < 0.001).

### Pointwise analysis

**Fig. 4:**
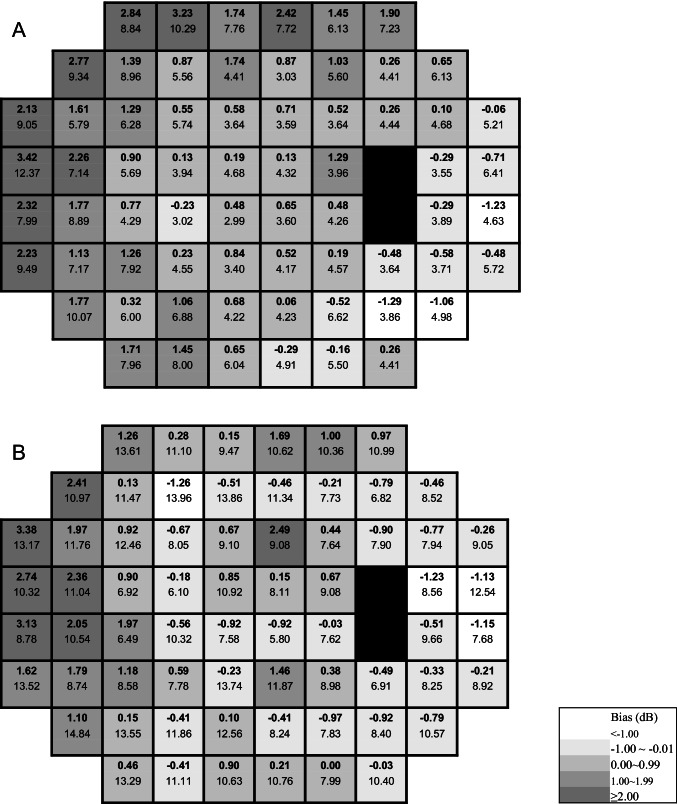
Pointwise analysis for mean sensitivity in the glaucoma (**A**) and cataract (**B**) groups

Pointwise between-strategy comparison using Bland–Altman agreement analysis was conducted for each of the corresponding 66 test points for each patient group separately (see Fig. 4 A, glaucoma patients; B, cataract patients). In both groups, the pointwise analysis showed a marked difference between superior temporal and inferior nasal fields, where SPARK Precision produced higher threshold estimates than SITA Standard in the nasal field compared to the temporal field.

### Between-strategy comparison of AGIS score

**Fig. 5:**
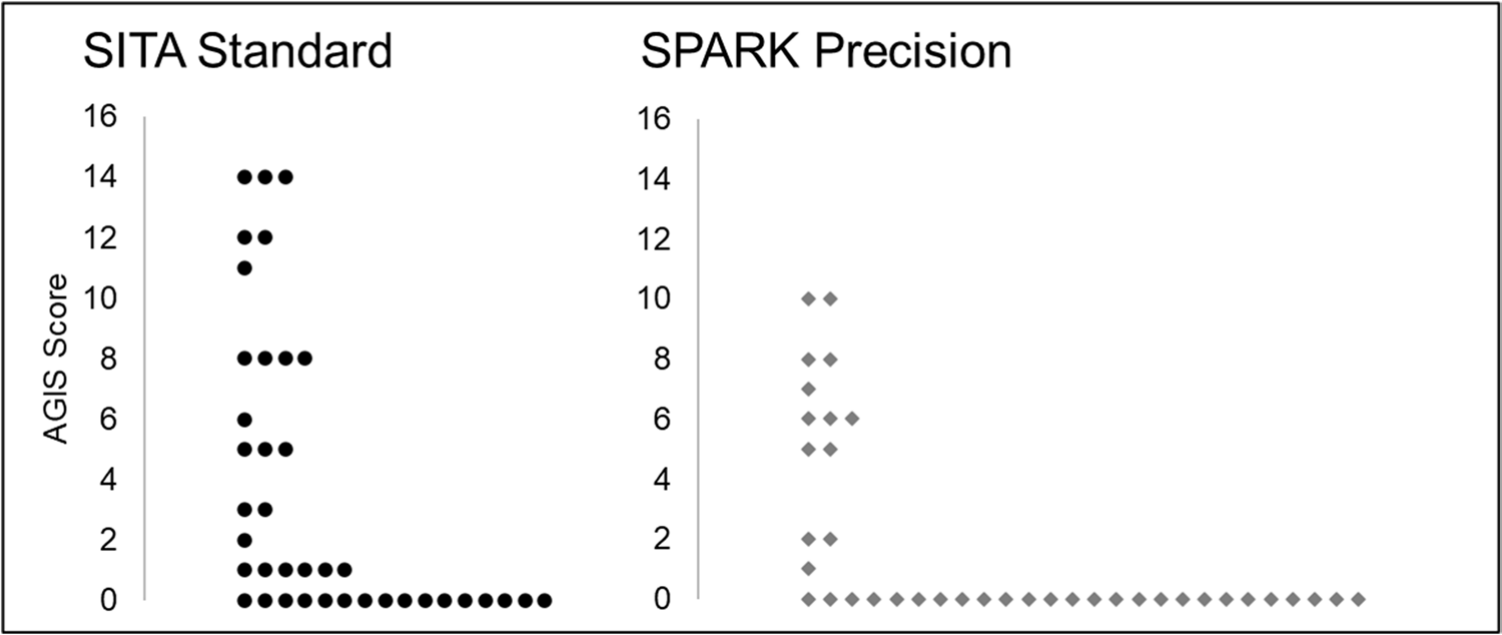
Severity of glaucomatous visual field loss defined by the AGIS score for SITA Standard and SPARK Precision

**Fig. 6:**
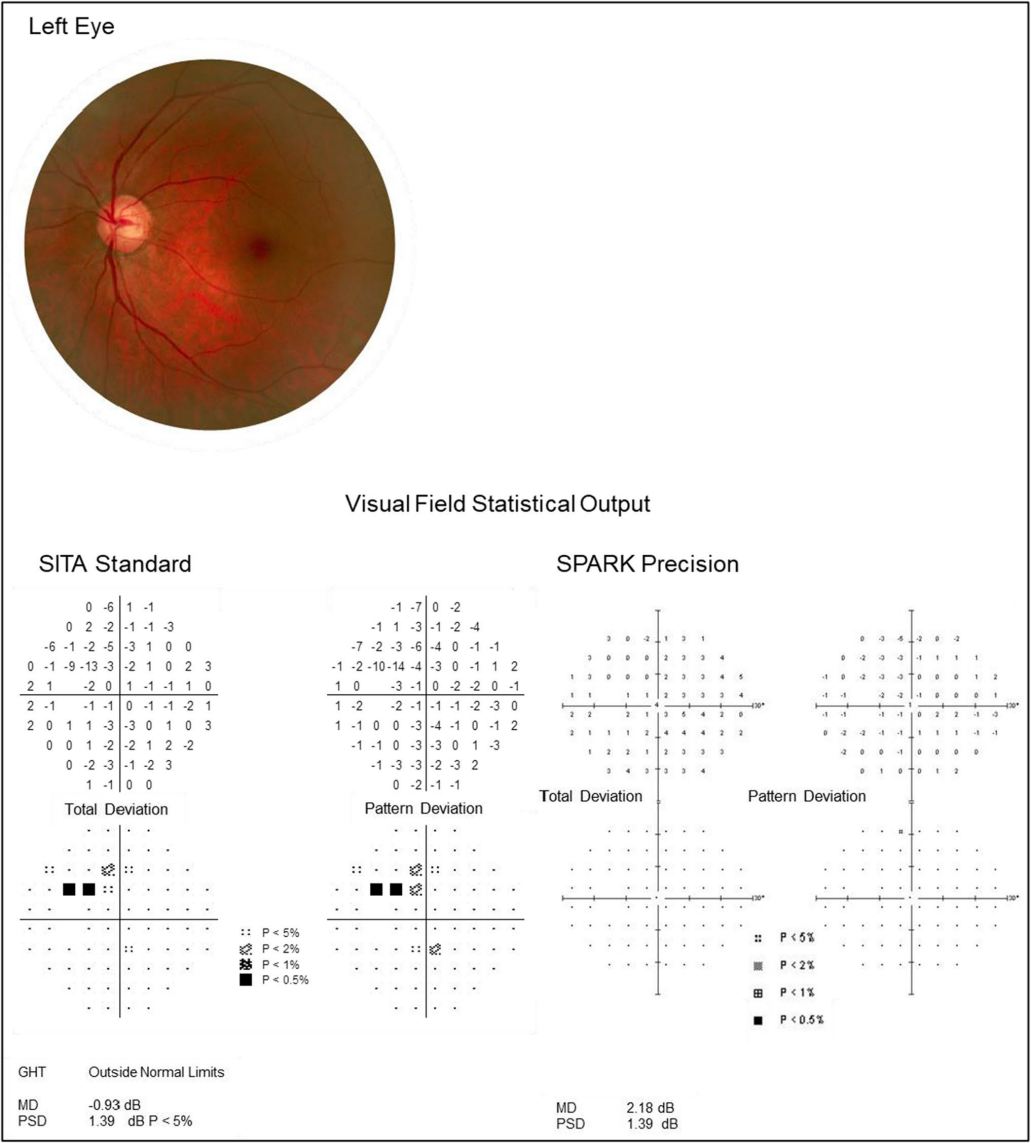
Illustrative example of a 69-year-old female glaucoma patient. SITA standard shows focal visual field loss and a glaucoma hemifield test outcome of ‘outside normal limits’. SPARK Precision shows an outcome of a normal visual field (with a ~ 3 dB more positive MD; interestingly, the PSD is identical)

The AGIS score was calculated for each glaucoma patient’s threshold results as measured by SITA Standard and SPARK Precision separately. A comparison of AGIS scores between the two thresholding methods is displayed in Fig. 4. There was a statistically significant difference between AGIS scores of the two thresholding methods (Table 5 and Figs. 5 and 6; Wilcoxon signed rank test: *Z* =  − 3.767, *p* < 0.001). Nevertheless, the AGIS scores from both strategies were highly correlated (Spearman correlation coefficient: rho =  − 0.750, *p* < 0.001).

**Table 5:** AGIS score using SITA Standard and SPARK Precision in glaucoma patients

	AGIS score				
	Mean	SD	Median	Min	Max
SITA Standard	3.69	4.76	1.00	0	14
SPARK Precision	1.96	3.23	0.00	0	10

## Discussion

Previous investigations in healthy subjects revealed a higher MS in SPARK Precision compared to SITA Standard [13, 21]. In this study of glaucoma and cataract patients, the MS of SPARK Precision was on average 1 dB higher than for SITA Standard. Lorch et al. suggest that this higher MS value may be due to a higher start value in SPARK. Indeed, threshold responses in SPARK Precision are interpolated from acquired thresholds at limited stimulus locations whereas each stimulus location in SITA is derived from independent assays at each stimulus location and therefore is influenced more by subject variability [13].

These factors may have resulted in an underestimation of focal (as represented by glaucomatous loss) and diffuse loss (as represented by cataract patients). Interestingly, the differences in MS between methods (see Fig. 4) appear to be asymmetrically distributed across the visual field with the greatest disparity in the superior nasal field and the lowest in the inferior temporal field. This demonstrates that there must be differences between threshold determination methods in SITA Standard and SPARK Precision.

**Fig. 7:**
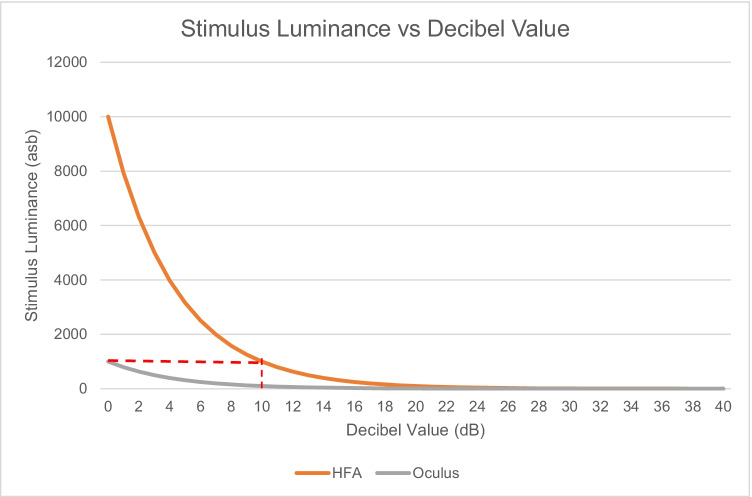
Decibel values and corresponding stimulus luminance for the HFA 3 and the Oculus Twinfield 2 perimeters

Overall, these findings have clinical implications, because in this sample of glaucoma patients, the severity of glaucomatous visual field loss measured with SPARK Precision was lower than that measured with SITA Standard. The AGIS scoring system for glaucoma was chosen because it represents glaucomatous visual field loss on a continuous scale as well as providing a severity classification [10]. These results show that SPARK underestimates the severity of glaucoma across our sample. This may be partly explained by the technical and methodological specifications of the Oculus Twinfield 2 perimeter. The measurement range of a perimeter is governed by the decibel scale, which typically ranges from 0 to 40 dB which is the range of typical human vision [22], according to the equation

1 $$dB=k+10\mathrm{log}\left(\frac{L}{\Delta L}\right)$$

where *L* is the background luminance in asb, ∆L is the stimulus luminance and *k* is a constant (40 for the HFA and 30 for the Oculus Twinfield 2); 0 dB represents the maximum stimulus luminance of a perimeter, which is 10,000asb in the HFA and 1,000asb in the Oculus Twinfield 2. Because both perimeters present their stimuli against a background luminance of 31.5asb, this means that when the Oculus reaches a threshold sensitivity of 0 dB, this is equivalent to 10 dB on the HFA (Fig. 7).

Consequently, the HFA is able to measure deeper defect depth than the Oculus Twinfield 2 perimeter. Both the Oculus Centerfield 2 and the Twinfield 2 perimeters have a maximum stimulus luminance of 1,000 asb, whereas the Oculus Easyfield 2 and Smartfield perimeters have a maximum stimulus luminance of 10,000 asb. All of the HFA and Oculus perimeters present stimuli onto a background luminance of 31.5 asb [23, 24]. Hence, the Centerfield 2 and Twinfield 2 perimeters have been manufactured with a reduced dynamic range compared to other Oculus perimeters and the HFA.

Because the Oculus Twinfield 2 has a reduced dynamic range compared to the HFA 3, it cannot quantify severe glaucomatous loss as defined by the AGIS scoring system. This is highlighted by our observation that none of the glaucomatous patients scored above 10 with AGIS in SPARK Precision compared to a maximum score of 14 in SITA Standard (Fig. 5). This study also found cases of glaucoma which were confirmed by an ophthalmologist that showed clear optic nerve head damage and corresponding early glaucomatous visual field loss on the HFA using SITA Standard, whilst the SPARK Precision probability plots were normal (illustrated by Fig. 6). This underestimation of focal visual field loss may be attributed to the higher starting threshold [21] as well as the influence of the neighbouring test points’ sensitivity in the final threshold estimate at a given location determined by SPARK Precision, which may be due to the fact that its development is based on TOP which has been found to result in higher sensitivity values, underestimation of the slope of visual field properties and underestimation of the severity of damage [25]. Interestingly in this patient, their MD (as determined by SPARK Precision) was 3 dB greater than SITA Standard and this may have contributed to the underestimation of focal loss.

In conclusion, the reduced dynamic range of the Oculus Twinfield 2 perimeter compared to the HFA and the methodology it employs to determine individual thresholds suggest that this perimeter and the threshold algorithm SPARK Precision cannot quantify early glaucomatous field loss or deeper scotomas due to the plateau effect caused by the reduced dynamic range.

## References

[1] Bengtsson B, Olsson J, Heijl A, Rootzén H (1997). A new generation of algorithms for computerized threshold perimetry. SITA Acta Ophthalmol Scandinavia.

[2] Bengtsson B, Heijl A, Olsson J (1998). Evaluation of a new threshold visual field strategy, SITA, in normal subjects. Acta Ophthalmol Scandinavica.

[3] Begtsson B, Heijl A (1998). Evaluation of a new perimetric strategy, SITA, in patients with manifest and suspect glaucoma. Acta Ophthalmol Scandinavica.

[4] OlssonJ BB, Heijl A, Rootzen H (1997). An improved method to estimate frequency of false positive answers in computerized perimetry. Acta Ophthalmol Scandinavica.

[5] King-Smith PE, Grigsby SS, Vingrys AJ, Venes SC, Supowit A (1994). Efficient and unbiased modifications of the Quest Threshold method: theory, simulations, experimental evaluation and practical implementation. Vision Res.

[6] Vingrys AJ, Pianta M (1999). A new look at threshold estimation algorithms for automated static perimetry. Optom Vis Sci.

[7] Turpin A, McKendrick AM, Johnson CA, Vingrys AJ (2002). Performance of efficient test procedures for frequency doubling technology in normal and glaucomatous eyes. Invest Ophthalmol Vis Sci.

[8] Turpin A, Mc Kendrick AM, Johnson CA, Vingrys AJ (2002). Development of efficient threshold strategies for frequency doubling technology perimetry using computer simulation. Invest Ophthalmol Vis Sci.

[9] Gonzalez de la Rosa M, Gonzalez-Hernandez M (2013). A strategy for averaged estimates of visual field threshold: Spark. J Glaucoma.

[10] Advanced Glaucoma Intervention Study 2 (1994). Visual field test scoring and reliability. Ophthalmology.

[11] Hodapp E, Parrish RK, Anderson DR (1993). Clinical decisions in glaucoma.

[12] Edgar D, Rudnicka A (2007). Glaucoma identification and co-management.

[13] Foo SK, Cubbidge RP, Heitmar R (2020). Comparison between two fast threshold strastegies: SPARK and SITA in normal subjects. Eur J Ophthalmol.

[14] Chylack LT, Wolfe JK, Singer DM, Leske MC, Bullimore MA, Bailey IL, Friend J, McCarthy D, Wu SY (1993). The lens opacities classification system III; the longitudinal study of cataract study group. Arch Ophthalmol.

[15] Karabassi M, Khu PM, Singer DM, Chylack LT (1993). Evaluation of lens opacities classification system III applied at the slit lamp. Optom Vis Sci.

[16] Brenton RS, Phelps CD (1986). The normal visual field on the Humphrey Field Analyzer. Ophthalmologica.

[17] de la Rosa MG, Gonzalez-Hernandez M, Sanchez-Garcia M (2013). Oculus-spark perimetry compared with 2 procedures of glaucoma morphologic analysis (GDx, HRT, and OCT). Eur J Ophthalmol.

[18] Garway-Heath DF, Poinoosawmy D, Fitzke FW, Hitchings RA (2000). Mapping the visual field to the optic disc in normal tension glaucoma eyes. Ophthalmology.

[19] González De La Rosa M, González Hernández M, Aguilar Estévez J, Abreu Reyes A, Pareja Ríos A (2002). Clasificación topográfica del campo visual glaucomatoso. Arch Soc Esp Oftalmol.

[20] Heijl A, Lindgren G, Olsson J (1989). The effect of perimetric experience in normal subjects. Arch Ophthalmol.

[21] Lorch L, Dietrich TJ, Schwabe R, Schiefer U (2001). Comparison of local differential luminance sensitivity (dls) between Oculus Twinfield Perimeter and Humphrey Field Analyzer 630 (HFA I) in normal volunteers of varying ages. Klin Monbl Augenheilkd.

[22] Heijl A, Patella VM, Bengtsson B. Effective perimetry. The field analyzer primer fourth edition

[23] https://www.oculus.de/en/products/perimetry/twinfield-2/technical-data/#produkte_navi. Accessed 03 Aug 2021

[24] https://www.zeiss.com/meditec/us/product-portfolio/perimetry/humphrey-visual-field-analyzer-3-with-sita-faster.html#downloads. Accessed 03 Aug 2021

[25] Anderson AJ (2003). Spatial resolution of the tendency-oriented perimetry algorithm. Invest Ophthalmol Vis Sci.

